# Editorial: Complement in the Development and Regeneration of the Nervous System

**DOI:** 10.3389/fimmu.2021.694810

**Published:** 2021-05-10

**Authors:** Faith H. Brennan, Liam G. Coulthard, Ali M. Alawieh, Orly Reiner, Marcela Pekna

**Affiliations:** ^1^ Department of Neuroscience, Center for Brain and Spinal Cord Repair, Belford Center for Spinal Cord Injury, The Ohio State University Wexner Medical Center, Columbus, OH, United States; ^2^ Royal Brisbane and Women’s Hospital, Herston, QLD, Australia; ^3^ Faculty of Medicine, University of Queensland, Herston, QLD, Australia; ^4^ Department of Neurosurgery, Emory University School of Medicine, Atlanta, GA, United States; ^5^ Department of Molecular Genetics, Weizmann Institute of Science, Rehovot, Israel; ^6^ Department of Clinical Neuroscience, Institute of Neuroscience and Physiology, Sahlgrenska Academy at the University of Gothenburg, Gothenburg, Sweden

**Keywords:** innate immune system, opsonization and clearance, anaphylatoxin receptors, membrane attack complex, demyelination and neuronal damage, neurodegenerating diseases, central nervous system, visual system

The complement system is an evolutionarily ancient arm of the innate immune system. It is composed of over 40 proteins, receptors and regulators that interact in a cascade manner to protect the host against pathogens (1). The soluble circulating complement proteins are mainly produced by hepatocytes. However, it is now well established that complement factors are expressed throughout the body, including in the central nervous system (CNS). In recent years, complement factors have been shown to control major aspects of CNS development, health, injury and disease (2–4). This Research Topic gathers the latest contributions to how complement factors interact with the nervous system, providing new mechanistic insight into neurodevelopment, cognitive function, myelination, and CNS infection. Review articles highlight roles for complement in development and degeneration of the visual system, and also the status of translation and clinical trials for complement-targeted therapeutics.

Using mice genetically deficient in C3aR (C3aR^-/-^), Pozo-Rodrigálvarez et al. identified new roles for complement C3a receptor (C3aR) in brain development. C3aR is expressed on neurons and glia throughout the brain and spinal cord, but its role in development has not been fully understood. This paper demonstrates that C3aR^-/-^ mice have abnormalities in the organization and morphology of the adult neocortex, amygdala, and hippocampus. These structural abnormalities are associated with motor hyperactivity and altered cognitive functions including short-term memory deficits, expanding on previous findings of impaired adult neurogenesis and memory deficits in C3aR^-/-^ animals (5, 6). These findings have important implications for understanding the cellular and molecular mechanisms that may underlie human neurodevelopmental disorders such as schizophrenia and attention deficit hyperactivity disorder.

Research by Tatomir et al. explored the intracellular signaling mechanisms triggered by sublytic levels of the complement membrane attack complex, C5b-9, on oligodendrocytes (OLGs). The classical role of C5b-9 is lysis of bacterial cells, but sublytic amounts of C5b-9 also decorate neurons and glial cells to control physiological and pathological processes. Tatomir et al. show that newly differentiated OLGs exposed to sublytic C5b-9 increase expression of NT2/CSPG4, a marker for OLG precursor cells (OPCs), and initiate cell cycle activation by inducing SIRT1 through c-jun, protein kinase C, and Pi3K/Akt signaling pathways. Sublytic C5b-9 also decreases expression of mature OLG markers, e.g. myelin basic protein. This resulting immature OLG phenotype that resembles OLG precursors may promote OLG survival in neuro-inflammatory environments and provide an opportunity for the cells to remyelinate axons after the inflammatory environment subsides. Harnessing this function of complement for clinical use may have implications for multiple sclerosis or other chronic demyelinating pathologies.

Shinjyo et al. characterized changes in expression of complement factors using an *in vivo* mouse model and *in vitro* primary glial cell cultures replicating *Toxoplasma gondii* infection. The study found that *Toxoplasma* triggers expression of genes coding for complement factors C3 and C1q, C3aR and C5aR1, properdin (CFP) and factor B (CFB) two weeks after infection, with CFP, CFB, C3aR and C5aR1 remaining elevated 8 weeks post-infection. Culture experiments demonstrate that the *Toxoplasma*-induced complement expression is at least partly driven by microglia. These data suggest that infection-induced complement activity is not solely confined to combatting the initial infection but may also affect the outcome of cerebral toxoplasmosis.

The articles reviewed by Borucki et al. discuss the role of complement in the development of the visual system and in its degeneration in various disorders, including multiple sclerosis, glaucoma, and age-related macular degeneration. This review updates readers on progress that has been made since the seminal work demonstrating the role of complement in refinement of the neural networks during development and degeneration of the visual pathway from the retina to the primary visual cortex (7). It highlights functional complexities of complement in the visual system, including neuroprotective actions, such as tagging myelin and other cellular debris for phagocytic removal, and also neurodegenerative aspects, including opsonization of stressed neurons and amplification of local inflammation. This review helps answer complex questions including: What complement-modulating strategies, systemic and targeted, have been used successfully in preclinical and clinical studies in the visual system? A deeper understanding of the mechanistic roles of complement in different diseases and stages of pathology will assist in the design and application of complement-targeted therapeutics for visual system disorders.

The review by Mallah et al. highlights major developments in anti-inflammatory and complement-targeted therapeutics for select neurological diseases, including stroke, traumatic brain injury, neuromyelitis optica, amyotrophic lateral sclerosis, multiple sclerosis, and Parkinson’s disease. This review answers common questions for the novice and expert reader about the status of complement therapeutics in CNS disease. It addresses the latest questions in complement therapeutics, including: which complement factors are optimal candidates for targeted therapy without significant off-target complications? Can currently approved complement-targeting drugs be repurposed for similar neurological disorders? What are the different strategies to fine-tune the pro-inflammatory and anti-inflammatory effects of complement in different CNS disease states?

We hope that this Research Topic provides readers with a useful reference for the state-of-the-art in CNS complement biology. Complement activation has been implicated in all major areas of CNS health and pathology, and plays dual roles in inflammation and repair. Therefore, we anticipate that research investigating complement engagement in the nervous system will continue to be a flourishing field for years to come, with targeted modulation of complement actions having the potential to alter the course of CNS disease.

## Author Contributions

All authors listed have made a substantial, direct and intellectual contribution to the work, and approved it for publication.

## Funding

FB is supported by the Wings for Life Spinal Research Foundation.

## Conflict of Interest

The authors declare that the research was conducted in the absence of any commercial or financial relationships that could be construed as a potential conflict of interest.
